# The Impact of Hematological Indices on the Occurrence of Delayed Graft Function (DGF) of Transplanted Kidney

**DOI:** 10.3390/jcm12247514

**Published:** 2023-12-05

**Authors:** Ewa Pilichowska, Piotr Ostrowski, Jerzy Sieńko

**Affiliations:** 1 Department of General Surgery and Transplantation, Pomeranian Medical University, 70-111 Szczecin, Poland; 2 Department of Nursing, Pomeranian Medical University, Żołnierska 48, 71-210 Szczecin, Poland; 3 Institute of Physical Culture Sciences, University of Szczecin, 70-453 Szczecin, Poland; jsien@poczta.onet.pl

**Keywords:** Delayed Graft Function (DGF), kidney transplantation, haematological indices

## Abstract

Background: to analyse the effect of haematological indices on the occurrence of Delayed Graft Function (DGF) in patients undergoing kidney transplantation and on the function of the transplanted kidney on the 7th postoperative day. Methods: 365 recipients who underwent kidney transplantation from a donor with known brain death between 2010 and 2017 were included in this retrospective study. Information from patient medical records, donor medical records, and donation and transplantation protocols was used for analysis. Statistica 13 was used for statistical analysis. Results: In the study group, DGF occurred in 144 recipients (39.45%), and Non-Graft Function (NGF) occurred in 12 recipients (3.29%). Recipients who developed DGF had a significantly higher Neutrophil/Monocyte Ratio (NMR) before renal transplantation (*p* = 0.048), a lower NMR value on postoperative day 1 (*p* < 0.001), and a difference between the values on day 1 and before surgery (*p* < 0.001). In addition, they had a significantly lower Lymphocyte/Monocyte Ratio (LMR) on postoperative day 1 LMR 1 (*p* < 0.001). It was shown that the value of the indices based on the ROC curve—NMR1 > 29.29, NMR1-0 > 22.71, and LMR1 > 1.74 (respectively: AUC = 0.624; 95% CI 0.566–0.682; and *p* < 0.001/AUC = 0.622; 95% CI 0.563–0.680; and *p* < 0.001/AUC = 0.610; 95% CI 0.550–0.670; and *p* < 0.001)—can be used to identify recipients with a significant probability of DGF. Conclusions: the NMR and LMR parameters on the first postoperative day and the difference between the NMR values on the first post-transplant day and the first pre-transplant day are predictive factors associated with the risk of DGF.

## 1. Introduction

The best form of renal replacement therapy is kidney transplantation. The immediate function of the transplanted kidney occurs in about half of the recipients, while in the remaining individuals, the function of the transplanted kidney may progressively appear, in which case we speak of Delayed Graft Function (DGF), the incidence of which is lowest after transplantation of a kidney from a living donor (4–10%) [1] and increases up to 53–69% after transplantation of a kidney from a DCD (Donation after Cardiac Death) donor [2]. The risk of DGF can be influenced by a variety of both immunologic and non-immunologic factors, the most important of which are as follows: donor age, cold and warm ischemia times, HLA (Human Leukocyte Antigen) compatibility, the immunosuppressive induction treatment, and the duration of dialysis therapy [3,4,5]. Algorithms have been created to estimate the risk of DGF, including the Irish nomogram [6], the Jeldres DGF nomogram [7], the Irish nomogram DGF calculator [8], and DGFS [9]. Common factors in the aforementioned algorithms are donor age and cold ischemia time. Indicators that have been developed are PLR (Platelets/Lymphocyte Ratio), NLR (Neutrophil/Lymphocyte Ratio), LMR (Lymphocyte/Monocyte Ratio), NMR (Neutrophil/Monocyte Ratio), and SII (Systemic Immune-Inflammation Index), which do not require additional laboratory tests besides a blood count with a smear. Most studies have used their value for prognostic evaluation in patients with cancer [10,11], cardiovascular disease, and renal failure [12,13,14]. One can find isolated studies describing the relationship between NLR, PLR, LMR, and SII and the function of the transplanted kidney [15,16,17,18]. To date, the effect of NMR on the function of the transplanted kidney has not been described.

## 2. Materials and Methods

The study included 365 recipients who underwent kidney transplantation between 2010 and 2017 at the Department of General and Transplant Surgery, SPSK2, Szczecin. The inclusion criteria for the study were organ transplantation from a donor with known brain death and laboratory tests performed on recipients before surgery and in the postoperative period at the hospital’s Laboratory Diagnostics Department. A retrospective analysis was performed based on information contained in patients’ hospital records, donor cards, and donation and transplantation protocols. The Bioethics Committee issued Certificate No. KB.006.30.2022/Z-3118, indicating that this study does not require its opinion. 

Whole blood was collected for laboratory tests before renal transplantation and on postoperative days 1 and 7. The parameters assessed were creatinine concentration (mg/dL), eGFR value (mL/min/1.73 m^2^; calculated according to the MDRD formula), the absolute number of neutrophils, lymphocytes, platelets, and monocytes, as well as the values of PLR (Platelets/Lymphocyte Ratio), NLR (Neutrophil/Lymphocyte Ratio), LMR (Lymphocyte/Monocyte Ratio), NMR (Neutrophil/Monocyte Ratio), and SII (Systemic Immune-Inflammation Index) indices. The indices are calculated as follows:

- NMR = Absolute Neutrophil Count (ANC)/Absolute Monocyte Count (AMC)
- PLR = Absolute Platelets Count (AMC)/Absolute Lymphocyte Count (ALC)
- NLR = Absolute Neutrophil Count (ANC)/Absolute Lymphocyte Count (ALC)
- LMR = Absolute Lymphocyte Count (ALC)/Absolute Monocyte Count (AMC)
- SII = NLR value × platelet count

Demographic and clinical data included age, sex, BMI, cause of renal failure, peak PRA (panel-reactive antibodies), number of HLA incompatibilities, cold ischemia time (CIT), warm ischemia time (WIT), type of renal replacement therapy, and induction therapy used. DGF occurred in 144 recipients, and Non-Graft Function (NGF) occured in 12 recipients.

Receiver operating characteristics Statistica 13 was used for statistical analysis. The Shapiro–Wilk test was performed to assess whether the data had a normal distribution. Non-parametric tests for independent variables—the Mann–Whitney U test in the case of two groups—were chosen to compare results between groups because of the lack of a normal distribution. ROC curves were drawn to indicate the factors influencing the possibility of DGF. A value of 0.05 was used as the level of significance. In this study, the need for dialysis therapy within seven days after organ transplantation was defined as DGF.

## 3. Results

The group of recipients consisted of 162 women and 203 men, aged 17 to 77 years. A description of the basic demographic and clinical data of the recipients is presented in Table 1A,B.

Kidneys were obtained from 224 donors with brain death. The donor was characterised using the following parameters: age, BMI, final creatinine concentration, and eGFR value (Table 2).

The recipients were divided into two groups according to the occurrence of DGF: no—recipients without DGF—and yes—recipients with DGF. The Mann–Whitney U test was performed to compare the parameters between the groups. Based on the analysis, recipients with delayed renal function had significantly longer haemodialysis time (*p* < 0.001) and CIT (*p* = 0.007), shorter peritoneal dialysis time (*p* = 0.001), a greater number of HLA mismatches (*p* = 0.01), a higher peak PRA (*p* = 0.015), monocyte counts before surgery (*p* = 0.028) and on postoperative day 1 (*p* < 0.001), NMR before transplantation (*p* = 0.048), and NMR and LMR on postoperative day 1 (*p* < 0.001) (Table 3 and Table 4).

The difference in their values was calculated to test whether the change in the values of haematological indices on postoperative day 1 compared to the values before renal transplantation had an impact on the occurrence of DGF. Statistical analysis showed a significantly lower difference in the group of recipients with DGF than in recipients without DGF (*p* < 0.001) (Table 4).

Next, ROC curve analysis was performed to determine whether the analysed parameters had a random effect on the risk of DGF. These showed that five parameters could be used to identify recipients with a significant probability of DGF: monocyte count before and on postoperative day 1, NMR1 value, NMR1-0, LMR1, haemodialysis time, peak PRA, and the number of HLA mismatches. NMR before transplantation was not included in the table because of a lack of statistical significance. AUC (Area Under the Curve) = 0.557; *p* = 0.067 (Table 5).

## 4. Discussion

Delayed graft function is one of the main factors affecting the function of the transplanted kidney in the first year after organ transplantation [19,20] and increases the risk of acute rejection and graft loss [19,21,22,23]. Recipients who develop DGF require an average hospital stay of six days longer after renal transplantation, which, together with repeated haemodialysis sessions, increases hospitalisation costs by at least 10% [24]. The incidence of DGF has remained unchanged despite recent improvements in immunosuppressive treatments. In the present study, DGF occurred in 39.45% of the recipients. Based on the studies conducted thus far, it is only possible to isolate factors that increase the risk of delayed transplanted kidney function, the most important being donor age, cold and warm ischemia time, HLA compatibility, donor DBD (Donation after Brain Death), recipient BMI, donor weight, immunosuppressive induction treatment used, and duration of dialysis [3,4,5,25,26]. In most cases, the haematological indices described in the literature are used to assess the prognosis of patients with cancer or cardiovascular disease. To date, only a few studies have assessed their impact on transplanted kidney function, and most of these are related to the NLR and PLR [13,15,16,27,28,29]. The value of estimating the probability of DGF based on peripheral blood count has not yet been appreciated.

One of the main mechanisms leading to delayed renal function is ischemia-reperfusion injury (IRI). Primary ischemia of the organ contributes to damage to endothelial cells, which secrete reactive oxygen species and promote the release of inflammatory mediators and proteolytic enzymes, and during reperfusion. The most vulnerable cells are proximal tubule epithelial cells, which are more likely to undergo necrosis [30]. In addition, the innate immune system contributes to renal IRI by activating bone marrow and renal tubular endothelial and epithelial cells [31]. In animal model studies, activation of TLRs (Toll-like receptors), mainly TLR-2 and TLR-4, in renal tubular epithelial cells was shown to stimulate effector cells of the innate immune system, including neutrophils and monocytes/macrophages [32,33]. Using flow cytometry, the cells that settled in the transplanted kidney after IRI were identified. Within the first 30 min, infiltration via neutrophils, macrophages, and T and B lymphocytes occurred. Neutrophil and macrophage levels peak within 24–48 h and remain elevated for a minimum of 6 days, confirming their pathogenic and damaging role in IRI [34].

Based on this analysis, a correlation between the number of circulating monocytes in the peripheral blood before kidney transplantation and on postoperative day 1 and the occurrence of DGF was demonstrated. It was observed that monocyte counts above 0.71 G/L before transplantation and above 0.21 G/L on postoperative day 1 were associated with the risk of delayed transplant kidney function.

In the present study, the significant difference in monocyte counts of 0.5 with a decrease on postoperative day 1 may be related to the infiltration of monocytes in the transplanted kidney in response to concentrations of chemokines released as a result of ischaemia-reperfusion injury and their transformation into tissue macrophages, which produce pro-inflammatory cytokines involved in tissue damage [34]. Donor macrophages, which also secrete pro-inflammatory factors, are present in the transplanted kidney. A study in a rat model showed that 2 h after reperfusion, monocytes adhered to the renal microvessel wall, contributing to greater organ injury [35]. In their study, Buberci and Paydas showed that recipients with a faster deteriorating eGFR value who received basiliximab during induction and a negative history of DGF or an episode of acute rejection, among others, had higher leukocyte, neutrophil, and monocyte counts and a lower LMR before renal transplantation. Recipients with monocyte values above 0.75 G/L before surgery had worse renal function at long-term follow-up [36]. Similarly, Guillen-Gomez et al. showed an association between higher monocyte levels and worse renal function at two-year follow-up [37]. Monocyte infiltration is associated with acute allograft dysfunction [38]. In a study by Wyburn et al., a positive correlation was observed between the number of monocytes and the degree of acute rejection. They reported that during acute rejection, local production of cytokines and chemokines—including MCP-1 (Monocyte Chemoattractant Protein-1; monocyte chemoattractant protein-1), RANTES (Regulated on Activation, Normal T-cell Expressed and Secreted), MIP-α (Macrophage Inflammatory Protein-alpha), Migration Inhibitory, and Macrophage Migration Inhibitory Factor (MIF), among others—via lymphocytes and macrophages attract monocytes, which then migrate through the endothelium and infiltrate the renal parenchyma through diapedesis [39].

In a study involving 1,594,700 patients, a higher incidence of chronic kidney disease and progression to end-stage renal failure was observed for monocyte counts above 0.70 G/L [40]. In the present study, platelets and platelet-associated haematological indices were not shown to significantly affect the occurrence of DGF. Platelets are actively involved in IRI, mainly during the reperfusion phase, as endothelial damage causes their activation and adhesion at the site of injury; moreover, they participate in the formation of the leukocyte infiltrate, including the formation of platelet–leukocyte aggregates (mainly with neutrophils, monocytes, and T and B lymphocytes) in the damaged and inflammation-changed tissue [41,42,43]. In this study, a non-DGF-related decrease in the platelet count on postoperative day 1 was observed. In an experimental mouse model, Andonegui et al. showed that an increase in TLR-4 (Toll-like receptors; Toll-like receptor) expression is associated with thrombocytopenia [44]. TLR-4, as already mentioned, is one of the main receptors present in the renal tubular epithelium and is involved in the activation of the innate inflammatory response during IRI [32,33].

The analysis showed significant differences between the groups of patients with and without DGF in the NMR index values before surgery (*p* = 0.048), NMR and LMR on postoperative day 1 (*p* < 0.001), and NMR values before and after kidney transplantation (*p* < 0.001). In the group of recipients with DGF, the NMR and LMR values on post-transplant day 1 as well as the difference in NMR before and after surgery were significantly lower (a group with DGF: 27.34; 1.35; and 17.10 vs. without DGF: 37.10; 1.77; and 27.12, respectively), while for NMR before surgery, the difference between groups was 0.09 (a group with DGF = 10.2 vs. without DGF = 10.09). In a univariate analysis, parameters such as NMR < 29.29 and LMR < 1.74 on postoperative day 1 and differences in NMR values between postoperative day 1 and before <22.74 were predictive of DGF.

Analysis of the NMR values and the number of monocytes and neutrophils before and on postoperative day 1 associated with a higher probability of DGF showed a significant decrease in the number of circulating monocytes in the peripheral blood on day 1 and an increase in the number of neutrophils. The LMR value on postoperative day 1 was lower than that before surgery, which was associated with a decrease in the number of lymphocytes and monocytes in the peripheral blood. The decrease in lymphocyte count on postoperative day 1 may be related to the immunosuppressive treatment administered, primarily induction with mono- and polyclonal antibodies and steroids. The usefulness of NMR and LMR in distinguishing a group of patients at increased risk of DGF may be related to the differentiated role of neutrophils, monocytes, and lymphocytes during the immune response. In addition, the demonstrated relationship between the difference in postoperative day 1 and preoperative day 1 NMR values is of great importance, as it highlights the importance of monocytes and neutrophils in the initial inflammatory phase after organ transplantation, among other IRI backgrounds. Neutrophils, the most abundant leukocytes in the peripheral blood, are actively involved in the acute phase of IRI by, among other things, closing small vessels in the transplanted kidney [18] and releasing oxygen-free radicals and proteases [31].

Hogendorf et al. observed an association between a higher absolute lymphocyte count and LMR, a lower NLR and PLR, and worse renal function on day 21 after transplantation based on eGFR [17]. In contrast, Buberci and Paydas, in their study, demonstrated an association between lower LMR and faster deterioration of renal function [36]. An elevated NLR > 3.5 and PLR > 120 before renal transplantation, according to Baral et al., favours the occurrence of DGF [18]. Halazun et al. identified three parameters that increased the risk of DGF: CIT > 15 h, NLR > 3.5, and donor type [15].

A haematological indicator combining the two main indicators of inflammation, NLR and PLR, is the SII, which is the product of NLR and platelet values or PLR and neutrophil values. It was initially used as a prognostic marker in cancer patients [45]. Most studies linking SII to transplantation have been related to liver transplantation [46,47,48]. Only Halpern et al. showed a negative correlation between SII and the occurrence of DGF [49]. In our analysis, there was no correlation between the SII, NLR, PLR, and DGF. This may be related to the lack of an effect of neutrophils, lymphocytes, and platelets on DGF in this study.

The use of NMR and LMR values and monocyte counts before surgery and on day 1 can help clinicians identify patients at higher risk of DGF. Optimising treatment, even more so in patients at higher risk of DGF, carries a lower cost than prolonged hospitalisation and additional haemodialysis for patients with DGF or those associated with faster graft loss and re-entry into a haemodialysis program. Researchers are looking for biomarkers specific to high-risk DGF recipients; however, their use is associated with high additional costs. The method proposed in this study to identify recipients who may have delayed transplant kidney function is not associated with any additional costs because blood counts with smears are performed on every patient in the perioperative period.

The calculation of the haematological indices NMR and LMR and the check of the absolute monocyte count do not require any special equipment, making it a simple diagnostic tool. A multicentre study could be performed in the future, taking into account the limitations identified after the study, including the following: qualification of kidney recipients with NGF, change in immunosuppression within 7 days of kidney transplantation, lack of data on serum calcineurin inhibitor levels, failure to include reoperation in the postoperative period, qualification of both first-time and repeat allograft recipients, and lack of information on organ procurement from ECD (Expanded Criteria Donor).

In summary, the NMR and LMR parameters on the first postoperative day and the difference between the NMR values on the first post-transplant day and the first pre-transplant day are predictive factors associated with the risk of DGF.

## Figures and Tables

**Table 1:** The basic demographic and clinical data of the recipients.

**A**				
**Parameters**			**N**	
Sex	Females		162 (44.38%)	
	Males		203 (55.62%)	
Age			51.5 ± 12.46	
BMI (kg/m^2^)			25.15 ± 4.15	
Cause of kidney failure	ADPKD		44 (12.05%)	
	Congenital malformations		17 (4.66%)	
	Glomerulonephritis		119 (32.60%)	
	Hypertensive nephropathy		49 (13.42%)	
	Diabetic nephropathy		32 (8.77%)	
	Unknown		45 (12.33%)	
	Other		65 (17.81%)	
Dialysis technique	Haemodialysis (HD)		296 (81.10%)	
	Peritoneal dialysis (PD)		40 (10.95%)	
	HD+PD		20 (5.48%)	
	Pre-emptive transplantation		9 (2.47%)	
pPRA (peak PRA)			15 ± 25	
HLA incompatibilities ABDR			3 ± 1.2	
CIT (h)			18 ± 7	
WIT (min)			23 ± 7.26	
Induction Therapy	Basiliksimab		182 (49.86%)	
	Antithymocyte Globulin		23 (6.30%)	
	Without		162 (44.38%)	
**B**				
**Parameters**	**0 Day**	**1st Day**		**7th Day**
Creatinine (mg/dL)	7.56 ± 2.65	6.49 ± 2.46		3.49 ± 2.87
eGFR (mL/min/1.73 m^2^)	8.44 ± 3.88	10.57 ± 6.27		36.8 ± 28.86
Lymphocytes (G/L)	1.72 ± 0.77	0.55 ± 0.37		1.76 ± 1.03
Neutrophils (G/L)	5.08 ± 2.67	10.8 ± 4.3		6.05 ± 3.22
Monocytes (G/L)	0.63 ± 0.28	0.49 ± 0.38		0.75 ± 0.34
Platelets (G/L)	214.02 ± 82.97	181.15 ± 58.75		207.32 ± 74,67
NLR	4.22 ± 6.73	27.81 ± 29.01		5.77 ± 8.39
PLR	152.92 ± 111.67	455.66 ± 383.55		170.23 ± 156.65
NMR	10.13 ± 9.89	33.17 ± 22.69		9.89 ± 10
LMR	3.19 ± 2	1.6 ± 1.23		2.69 ± 2.04
SII	904.15 ± 1544.85	5030.51 ± 5216.99		1103.95 ± 1515.25

PRA—panel-reactive antibodies; CIT—cold ischemia time; and WIT—warm ischemia time. PLR—Platelets/Lymphocyte Ratio; NLR—Neutrophil/Lymphocyte Ratio; LMR—Lymphocyte/Monocyte Ratio; NMR—Neutrophil/Monocyte Ratio; and SII—Systemic Immune-Inflammation Index.

**Table 2:** The basic demographic and clinical data of the donors.

Parameters		N
Sex	Females	76 (33.93%)
	Males	148 (66.07%)
Age		47.55 ± 15.31
BMI (kg/m^2^)		25.81 ± 3.64
Last creatinine level (mg/dL)		1.04 ± 0.42
eGFR (mL/min/1.73m^2^)		86.88 ± 35.58

**Table 3:** Relationships between the occurrence of Delayed Graft Function and factors dependent on the recipient, donor, and perioperative parameters.

Parameters	DGF	N	Mean	SD	Mann–Whitney U Test
					*p* Value
**Donor-dependent factors**					
**Age**	No	221	46.49	15.472	0.238
	Yes	144	48.23	14.885	
**BMI (kg/m²)**	No	190	25.65	3.613	0.633
	Yes	124	25.55	3.372	
**Last Cr. level**	No	193	1.00	0.380	0.450
	Yes	128	1.07	0.47	
**eGFR (mL/min/1.73 m^2^)**	No	192	88.47	34.430	0.369
	Yes	128	84.03	34.182	
**Recipient-dependent factors**					
**Age**	No	221	51.63	12.473	0.808
	Yes	144	51.43	12.507	
**BMI (kg/m²)**	No	216	24.85	4.033	0.110
	Yes	143	25.64	4.266	
**PD** **(months)**	No	219	5.63	16.298	**0.001**
	Yes	143	1.66	6.514	
**HD** **(months)**	No	215	27.35	26.637	**<0.001**
	Yes	143	41.93	35.787	
**Peak PRA**	No	220	11.45	21.097	**0.015**
	Yes	144	19.97	29.842	
**PRA**	No	221	6.12	14.604	0.408
	Yes	143	11.44	23.219	
**HLA MM**	No	221	2.86	1.258	**0.01**
	Yes	144	3.22	1.07	
**Perioperative parameters**					
**CIT (h)**	No	193	16.73	7.153	**0.007**
	Yes	125	19.02	7.481	
**WIT (min.)**	No	183	22.90	6.817	0.061
	Yes	112	24.55	8.127	

SD—standard deviation; Cr—creatinine; HLA MM—number of HLA mismatches; PD—peritoneal dialysis; HD—haemodialysis; CIT—cold ischemia time; and WIT—warm ischemia time.

**Table 4:** Relationships between the occurrence of Delayed Graft Function and haematological indices.

Parameter	DGF	N	Mean	SD	Mann–Whitney U Test
					*p* Value
**Lymphocytes 0 (G/L)**	No	221	1.71	0.782	0.877
	Yes	144	1.73	0.767	
**Lymphocytes 1 (G/L)**	No	220	0.54	0.374	0.334
	Yes	143	0.58	0.374	
**Lymphocytes 7 (G/L)**	No	216	1.99	1.10	**<0.001**
	Yes	142	1.42	0.78	
**Neutrophils 0 (G/L)**	No	221	5.04	2.305	0.398
	Yes	144	5.11	3.195	
**Neutrophils 1 (G/L)**	No	220	10.63	4.070	0.341
	Yes	143	11.06	4.670	
**Neutrophils 7 (G/L)**	No	216	6.12	3.54	0.797
	Yes	142	5.94	2.66	
**Monocytes 0 (G/L)**	No	221	0.61	0.281	**0.028**
	Yes	144	0.66	0.286	
**Monocytes 1 (G/L)**	No	220	0.44	0.357	**<0.001**
	Yes	143	0.56	0.410	
**Monocytes 7 (G/L)**	No	216	0.75	0.33	0.873
	Yes	142	0.75	0.37	
**Platelets 0 (G/L)**	No	221	216.34	67.951	0.623
	Yes	144	211.16	62.674	
**Platelets 1 (G/L)**	No	220	183.35	59.991	0.321
	Yes	143	177.97	57.482	
**Platelets 7 (G/L)**	No	216	220.09	82.91	**<0.001**
	Yes	142	187.46	56.02	
**NLR 0**	No	221	4.10	5.903	0.431
	Yes	144	4.39	7.942	
**NLR 1**	No	220	25.85	18.197	0.912
	Yes	143	30.91	40.690	
**NLR Δ1-0**	No	220	21.78	18.894	0.892
	Yes	143	26.50	41.570	
**PLR 0**	No	221	156.59	120.088	0.751
	Yes	144	147.39	98.651	
**PLR 1**	No	220	452.28	341.643	0.297
	Yes	143	461.61	446.768	
**PLR Δ1-0**	No	220	296.40	340.153	0.408
	Yes	143	314.25	448.774	
**NMR 0**	No	221	10.11	8.011	**0.048**
	Yes	144	10.20	12.405	
**NMR 1**	No	220	37.10	24.576	**<0.001**
	Yes	143	27.34	18.111	
**NMR Δ1-0**	No	220	27.12	25.597	**<0.001**
	Yes	143	17.10	21.835	
**LMR 0**	No	221	3.18	1.763	0.307
	Yes	144	3.19	2.315	
**LMR 1**	No	220	1.77	1.302	**<0.001**
	Yes	143	1.35	1.062	
**LMR Δ1-0**	No	220	−1.41	1.813	0.079
	Yes	143	−1.84	2.241	
**SII 0**	No	221	913.08	1482.584	0.502
	Yes	144	890.32	1661.570	
**SII 1**	No	220	4746.57	3940.796	0.671
	Yes	143	5486.51	6790.670	
**SII Δ1-0**	No	220	3840.95	3895.964	0.934
	Yes	143	4592.95	6880.85	

PLR—Platelets/Lymphocyte Ratio; NLR—Neutrophil/Lymphocyte Ratio; LMR—Lymphocyte/Monocyte Ratio; NMR—Neutrophil/Monocyte Ratio; SII—Systemic Immune-Inflammation Index; SD—standard deviation; Δ1-0—difference in values on postoperative day 1 and before transplantation; 0—value before transplantation; 1—value on 1st postoperative day; and 7—value on 7th postoperative day.

**Table 5:** Logistic regression model summary for statistically significant predictors.

Parameters	AUC	*p* Value	Cut-Off Point	95% CI	Sensitivity	Specificity	Accuracy
Mono.0	0.566	**0.034**	0.71	0.505–0.628	0.458	0.697	0.603
Mono.1	0.623	**<0.001**	0.21	0.565–0.680	0.888	0.345	0.559
NMR1	0.624	**<0.001**	29.29	0.566–0.682	0.678	0.545	0.598
NMRΔ1-0	0.622	**<0.001**	22.74	0.563–0.680	0.734	0.514	0.601
LMR1	0.610	**<0.001**	0.790	0.550–0.670	0.790	0.409	0.559
**HD (month)**	0.653	**<0.001**	36	0.583–0.723	0.552	0.717	0.652
**Peak PRA**	0.583	**0.031**	20	0.507–0.658	0.313	0.823	0.621
**HLA MM**	0.578	**0.012**	3	0.521–0.634	0.750	0.384	0.529

AUC—Area Under the Curve; Mono.—monocytes; LMR—Lymphocyte/Monocyte Ratio; NMR—Neutrophil/Monocyte Ratio; HD—haemodialysis; HLA MM—number of HLA mismatches; Δ1-0—difference in values on postoperative day 1 and before transplantation; 0—value before transplantation; and 1—value on 1st postoperative day.

## Data Availability

Data are contained within the article.
